# Elevator fault precursor prediction based on improved LSTM-AE algorithm and TSO-VMD denoising technique

**DOI:** 10.1371/journal.pone.0320566

**Published:** 2025-04-24

**Authors:** Hao Cao, Xiaoyan Du

**Affiliations:** 1 School of Architecture and Engineering, Xuchang Vocational and Technical College, China; 2 Department of Electrical Engineering, Xuchang Electrical Vocational College, China; Sri Venkateswara University College of Engineering, INDIA

## Abstract

This study proposes an advanced elevator fault precursor prediction method integrating Variational Mode Decomposition (VMD), Bidirectional Long Short-Term Memory (BILSTM), and an Autoencoder with an Attention Mechanism (AEAM), collectively referred to as the VMD-BILSTM-AEAM algorithm. This model addresses the challenges of feature redundancy and noise interference in elevator operation data, improving the stability and accuracy of fault predictions. Using a dataset of elevator operation parameters, including current, voltage, and running speed, the model utilizes the Attribute Correlation Density Ranking (ACDR) method for feature selection and the TSO-optimized VMD for denoising, enhancing data quality. Cross-validation and statistical analyses, including confidence interval calculations, were employed to validate the robustness of the model. The results demonstrate that the VMD-BILSTM-AEAM algorithm achieves a mean True Positive Rate (TPR) of 0.919 with a 95% confidence interval of 0.915 to 0.924, a mean False Positive Rate (FPR) of 0.090 with a 95% confidence interval of 0.087 to 0.092, and a mean Area Under the Curve (AUC) of 0.919 with a 95% confidence interval of 0.915 to 0.923. These performance metrics indicate a significant improvement over traditional and other deep learning models, confirming the model’s superiority in predictive maintenance of elevators. The robust capability of the VMD-BILSTM-AEAM algorithm to accurately process and analyze time-series data, even in the presence of noise, highlights its potential for broader applications in predictive maintenance and fault detection across various domains.

## Introduction

Elevators, as essential vertical transportation devices, play a pivotal role in modern urban life. With the rapid acceleration of urbanization, the number of elevators has increased significantly, highlighting the critical need for effective fault prediction and maintenance strategies [1,2]. Traditional maintenance approaches, which rely on regular inspections or reactive repairs post-failure, are not only time-consuming and labor-intensive but also fail to predict faults proactively, thereby posing significant safety risks [3,4].

In recent years, the advent of artificial intelligence has led to growing interest in data-driven fault prediction methods. Pan et al. [5] developed an elevator risk evaluation method based on fuzzy theory and machine learning algorithms, establishing a risk evaluation index system for elevator operation. They employed a fuzzy comprehensive evaluation approach to assess the risk levels of 50 elevators and optimized a support vector machine (SVM) model using the maximum information coefficient and correlation-based feature selection (MIC-CFS) alongside the improved gray wolf algorithm (IGWO). Their findings demonstrated that integrating machine learning methods enhances efficiency and accuracy compared to traditional expert-based evaluations, with the optimized SVM showing faster convergence, greater stability, and higher accuracy. Qiu et al. [6] applied a Backpropagation (BP) neural network model optimized by the Modified Particle Genetic Algorithm (MPGA) to predict elevator door lock faults caused by external forces. Although the MPGA-BP algorithm mitigates the dependency on random weight initialization, there remains potential for enhancing model convergence speed and reducing training time. Wen et al. [7] used the Particle Swarm Optimization-BP (PSO-BP) algorithm to analyze the electrical drive of door lock contacts, simulating elevator door faults through vibration frequency analysis. While the PSO-BP algorithm addressed learning rate and convergence speed issues influenced by node numbers, it exhibited strong randomness and dependence on initial parameters. Zhang et al. [8] employed a deep convolutional forest neural network algorithm to analyze and predict elevator door faults caused by human factors, demonstrating improved accuracy and recall rates over traditional algorithms, but still faced challenges with slow model convergence and computational complexity.

Time series models, which inherently capture the temporal dependencies in data, are highly effective for predicting sequences with strong temporal correlations. Long Short-Term Memory (LSTM) networks are particularly adept at mining information from time series data due to their specialized memory units, while Autoencoders (AE) excel in extracting features from nonlinear data through their unique encoding and decoding structures. Iuculano et al. [9] explored the use of LSTM networks to predict safety-related elevator breakdowns that result in immediate stops, utilizing a balanced dataset from global elevator monitoring data. Their study demonstrated the LSTM model’s effectiveness in forecasting safety chain malfunctions, achieving an average F1-score of 85% across multiple time windows. Dai et al. [10] proposed an LSTM-AE model utilizing multi-layer LSTM networks as the encoding and decoding structures, enhancing the extraction of nonlinear temporal features. The LSTM-AE algorithm, with its robust feature extraction capabilities, has found extensive application in time series data analysis. Yu et al. [11] used the LSTM-AE algorithm for reconstructing mechanical system operation curves to predict system lifetimes, incorporating Bidirectional LSTM (BILSTM) to refine the encoding structure and enhance prediction accuracy. Similarly, Teng et al. [12] employed the LSTM-AE algorithm to capture time-dependent features of population inflows and outflows in travel demand prediction, integrating an attention mechanism to prioritize encoding hidden states relevant to passenger flow, thereby improving decoder input quality and overall prediction accuracy.

However, existing deep learning algorithms often require extensive datasets, and the presence of redundant features in the feature vectors can significantly degrade the algorithm’s accuracy and real-time performance. In real-world elevator operation and maintenance scenarios, the parameter set is typically a multivariate time series that includes voltage, current, frequency, acceleration, and running speed. The correlations among these variables introduce redundancy, complicating subsequent analysis [13]. Sun et al. [13] proposed an attribute selection algorithm based on mutual information and class separability to address feature redundancy by ranking attributes based on their redundancy, though this approach is computationally intensive due to its reliance on probability density functions. BACCIU [14] introduced an incremental mutual correlation filter-based attribute selection method, which calculates attribute redundancy via sliding dot product operations and sets a redundancy threshold to eliminate noise and redundant attributes; however, this method’s performance is highly dependent on the appropriate setting of the redundancy threshold, which often requires expert input. Zhao et al. [15] developed a filter feature selection algorithm called RPFMI, based on mutual information, to enhance intrusion detection efficiency by accounting for feature redundancy, class impact, and feature-class relationships, achieving impressive accuracy rates of 99.772% on DOS data and 97.749% on the Kyoto 2006 + dataset. Zhang et al. [16] proposed an attribute subset selection method based on attribute correlation density ranking (ACDR), which selects the most representative attribute subset by comparing the product of distinctiveness and representativeness, thus reducing computational complexity and reliance on expert judgment.

During elevator operation, parameters such as current and voltage generally remain stable, whereas passenger movements can introduce noise into the running speed curve, affecting prediction accuracy. Therefore, stabilizing and denoising the running speed series is essential. Various denoising techniques for non-stationary sequences, including Empirical Mode Decomposition (EMD), ensemble empirical mode decomposition, complementary ensemble empirical mode decomposition, and local mean decomposition, have been proposed. Although these methods achieve sequence stabilization through recursive decomposition, they are limited by issues such as mode mixing and endpoint effects. Variational Mode Decomposition (VMD) addresses these limitations by incorporating variational constraints, leading to improved denoising performance for non-stationary sequences. Nevertheless, VMD requires the manual setting of parameters, presenting challenges in optimal parameter selection. Zhang et al. [17] introduced the Tuna Swarm Optimization (TSO) algorithm to automate the optimization of VMD parameters, enhancing prediction accuracy. To tackle the challenges of feature redundancy and noise in predicting elevator fault precursors, this study employs the ACDR method for feature selection, effectively removing redundant information and combining it with the TSO-VMD method for stabilizing and denoising running speed data. By integrating a sliding window attention mechanism with BILSTM into the LSTM-AE model, the proposed approach significantly improves feature extraction from time series data, providing robust predictive maintenance capabilities for elevators.

## Method

**Fig 1:**
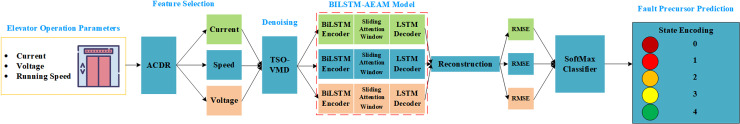
Schematic diagram of TSO-VMD-BILSTM-AEAM algorithm structure.

This section introduces the principles of the article’s model. The main approach involves using the ACDR algorithm and the VMD algorithm optimized by TSO for feature selection and denoising of elevator operation parameters. Based on the traditional LSTM-AE model, improvements are made to the model’s encoding network and decoder input structure, resulting in the TSO-VMD-BILSTM-AEAM algorithm, whose architecture is shown in Fig 1. First, the ACDR algorithm is used to eliminate redundant parameters in the original parameter set and determine the features required for predicting precursors. Next, the TSO-VMD algorithm is applied to perform modal decomposition on the running speed time series data, removing high-frequency noise from the speed sequence and achieving denoising and stabilization of the speed signal [18–20]. Then, the processed feature sequence is input into the BILSTM-AEAM model for reconstruction, with multiple BILSTM units encoding the time series data. A sliding window attention mechanism that integrates BILSTM is designed between the encoder and decoder to improve the quality of the decoder’s input, thereby enhancing the model’s ability to extract temporal features. Finally, the re-construction errors of each feature sequence are integrated through a SoftMax classifier to predict elevator fault precursors.

### ACDR method

To address the issue of feature redundancy in the original elevator parameter set, this paper uses the ACDR method to eliminate redundant elevator parameters and select features for elevator precursor prediction, such as current, voltage, and running speed [15–17].

The ACDR algorithm calculates the distinctiveness (*ρ*_*i*_) and representativeness(*δ*_*i*_) of each parameter based on the correlation coefficient (*d*_*ij*_) between parameters. The product of distinctiveness and representativeness (*r*_*i*_) is used as a measure to select the feature vector set by comparing the *r*_*i*_ values of each parameter. The formulas for calculating *ρ*_*i*_, *δ*_*i*_, and *r*_*i*_ are as follows:


$$\rho_{i}=\sum_{j=1,j\neq i}^{n}{\text{e}}^{\left(-d_{ij}^{2}/d_{c}^{2}\right)}$$ (1)

$$\delta_{i}=\underset{j:\rho_{j}>p_{i}}{\min}\left(d_{ij}\right)$$ (2)

$$r_{i}=\delta_{i}\times \rho_{i}$$ (3)

In the formula, dc is the cutoff distance. The selection criterion for dc is that the clustering effect is better when the number of neighbors for each sample point is about 1% to 2% of the total number of samples. In this paper, 2% is used to calculate dc.

**Fig 2:**
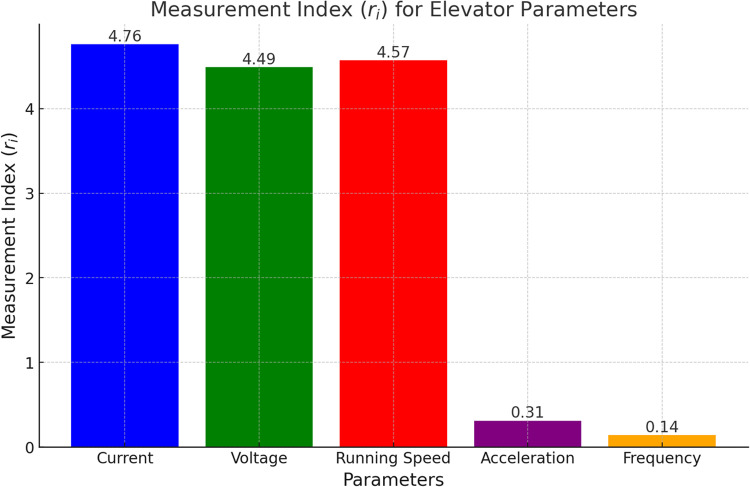
Measurement index diagram of elevator operating parameters.

The *r*_*i*_ values of each parameter are calculated using the ACDR algorithm, and the results are shown in Fig 2. In the Fig, the measurement indices *r*_*i*_ for current, voltage, and running speed are all above 4.4, while those for frequency and acceleration are below 0.4. The *r*_*i*_ values of the first three parameters are an order of magnitude higher than those of the latter two, indicating that they can represent the entire parameter set. Therefore, frequency and acceleration are eliminated from the original parameter set, and current, voltage, and running speed are selected as the feature vector set for four types of elevator faults, including overspeed current.

### VMD algorithm

The essence of the VMD algorithm is to decompose the signal x(t) into k modal components with certain sparsity through iterative search of the variational model, where the k components are within different frequency bandwidths. By selecting the optimal modal curve, a denoised and stable sequence is obtained. The VMD algorithm introduces Lagrange multipliers λ and quadratic penalty terms to determine the unconstrained problem, as shown below:


$$L\left(\left\{u_{k}\right\},\left\{\omega_{k}\right\},\lambda \right)=\alpha \sum_{k} \|\partial_{t}\left(\delta (t)+\frac{\text{j}}{\pi t}\right)\times \left.u_{k}(t)\right]{\left.{\text{e}}^{-\text{j}\omega_{k}t}\right\|}_{2}^{2}+{ \|\left.f(t)-\sum_{k}u_{k}(t)\right\|}_{2}^{2}+ \langle \lambda (t),f(t)-\sum_{k}u_{k}(t)\rangle$$ (4)

In the above formula, α is the penalty factor, *u*_*k*_ and *ω*_*k*_ are the k components of the variational mode decomposition and their corresponding frequency centers, respectively.

During the ascent or descent of an elevator, the running curves of current or voltage are relatively stable. However, behaviors such as children’s roughhousing or playing can cause the elevator to shake, resulting in high-frequency noise signals in the running speed time series data. When extracting features from a non-stationary sequence containing noise, the temporal information of the noise signals is learned, thereby reducing the effectiveness of feature extraction. In this paper, the proposed TSO-VMD algorithm is used as the denoising method for the speed sequence.

### Tuna swarm optimization

Tunas are carnivorous fish that live in the ocean and swim very fast, but still not as fast as their prey. Therefore, during hunting, tunas act collectively and employ two effective strategies for foraging. The first is spiral foraging, where they swim in a spiral motion to drive the prey into shallower waters where it is easier to attack [21–23]. The second is parabolic foraging, where each tuna follows the one in front of it, forming a parabolic shape to surround the prey.

The Tuna Swarm Optimization algorithm has a simple structure, requires few parameters to be adjusted, and is easy to implement. It starts by randomly generating N tuna individuals in a D-dimensional search space to form the initial population:


$$X_{i}^{\text{init}}=rand(ub-ub)+lb,i=1,2,\cdots ,N$$ (5)

In the formula, Xi represents the initial position of the *i*^*th*^ tuna individual; rand is a random vector uniformly distributed in the range (0,1); *ub* and *lb* are the upper and lower boundaries of the search space, respectively; and N is the number of individuals in the tuna swarm.

When the target is difficult to lock onto, tunas will pursue it in a dense spiral formation. During the pursuit, the tuna swarm will exchange information to enable sharing of information between adjacent individuals. The position update formula for spiral foraging is as follows:


$$X_{i}^{t+1}= \{\begin{array}{c} c_{1}\left(X_{\text{best}}^{t}+\beta \left|X_{\text{best}}^{t}-X_{i}^{t}\right|\right)+c_{2}X_{i}^{t},i=1\\ c_{1}\left(X_{\text{best}}^{t}+\beta \left|X_{\text{best}}^{t}-X_{i}^{t}\right|\right)+c_{2}X_{i-1}^{t},i=2,3,\cdots ,N \end{array}$$ (6)

$$c_{1}=a+(1-a)\frac{t}{t_{\max}}$$ (7)

$$c_{2}=(1-a)-(1-a)\frac{t}{t_{\max}}$$ (8)

$$\beta ={\text{e}}^{bl}\cos(2\pi b)$$ (9)

$$l={\text{e}}^{3\cos\left(\left(\left(t_{\max}+1/t\right)-1\right)\pi \right)}$$ (10)

In the formula, *X* represents the position of the individual in the (*t + 1*)^*th*^ iteration; $X_{\text{best}}^{t}$ is the current best individual position; *c*_*1*_ and *c*_*2*_ are weight coefficients; *t* represents the current iteration number; *t*_*max*_ is the maximum number of iterations; and *b* is a random number in the range (0,1).

When the optimal individual is unable to find food, a coordinate is randomly generated in the search space to serve as a reference point for spiral foraging.


$$X_{i}^{t+1}= \{\begin{array}{l} c_{1}\left(X_{\text{rand}}^{t}\beta \left|X_{\text{rand}}^{t}-X_{i}^{t}\right|\right)+c_{2}X_{i}^{t},\\ c_{1}\left(X_{\text{rand}}^{t}+\beta \left|X_{\text{rand}}^{t}-X_{i}^{t}\right|\right)+c_{2}X_{i-1}^{t},i=2,3,\cdots ,N \end{array}$$ (11)

In the formula, $X_{\text{rand}}^{t}$ represents a randomly generated coordinate point in the search space.

In addition to spiral foraging, tunas also engage in parabolic foraging, where they form a parabola with the food as the reference point. The tuna swarm searches around to locate the position of the food. The specific mathematical model is as follows:


$$X_{i}^{t+1}= \{\begin{array}{c} X_{\text{best}}^{t}+rand\left(X_{\text{best}}^{t}-X_{i}^{t}\right)+TF\cdot p^{2}\left(X_{\text{best}}^{t}-X_{i}^{t}\right),\text{if rand}<0.5\\ TF\cdot p^{2}X_{i}^{t},\text{if rand}<0.5 \end{array}$$ (12)

$$p={\left(1-\frac{t}{t_{\max}}\right)}^{\left(t/t_{\max}\right)}$$ (13)

In the formula, TF represents a random number that is either 1 or -1. Throughout the optimization process, the TSO algorithm continuously updates and calculates until the final condition is satisfied.

### TSO-VWD

**Fig 3:**
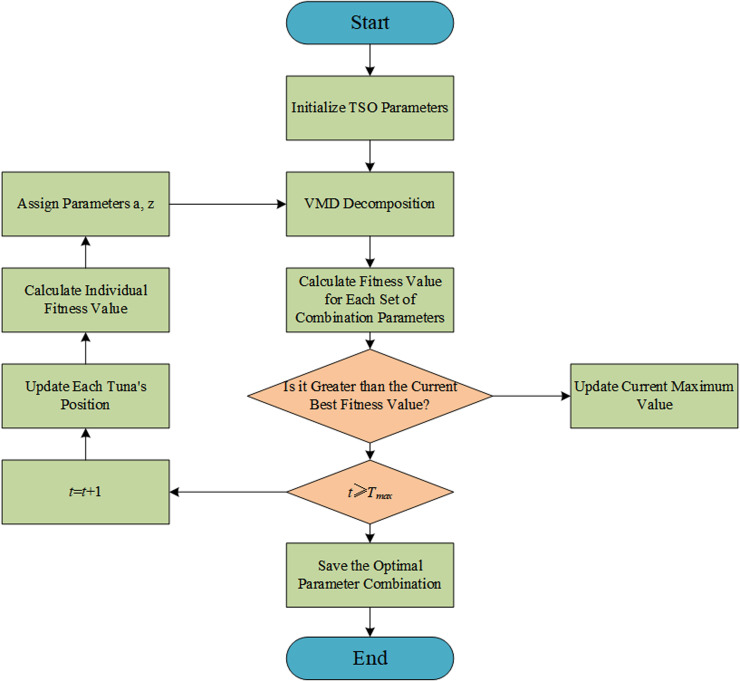
Diagram of TSO optimized VMD parameter.

**Fig 4:**
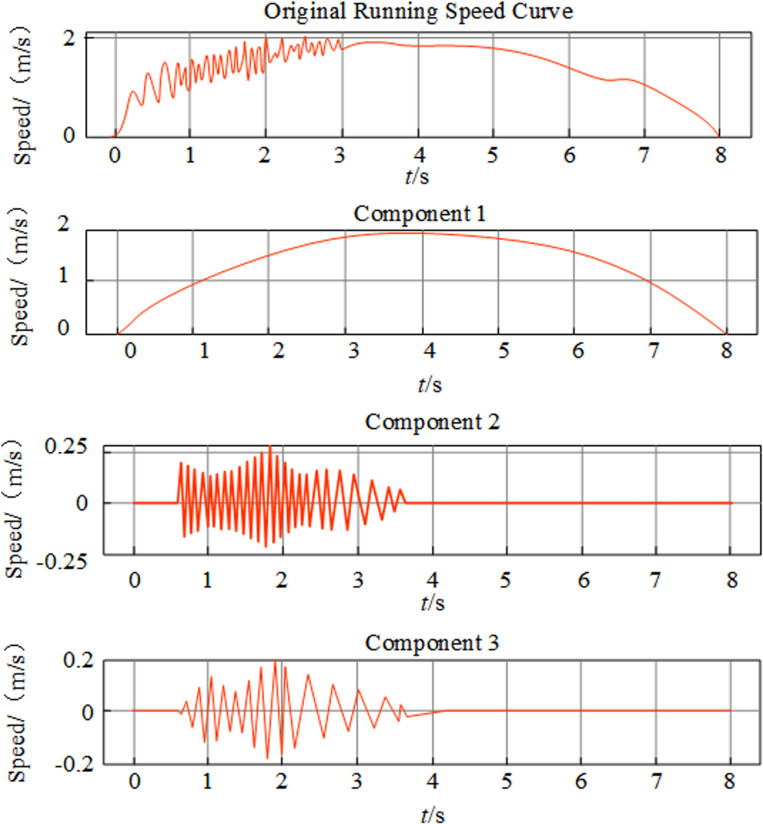
Variational modal decomposition diagram of running speed sequence.

In this paper, we take a piece of original running speed time series data containing noise as an example and automatically optimize the VMD parameters k and α using the TSO algorithm. The process is shown in Fig 3. The steps are as follows:

The Tuna Swarm Optimization (TSO) algorithm was applied to automatically optimize the parameters K (the number of modes) and α(the balancing parameter) of the Variational Mode Decomposition (VMD) for a noisy time series of elevator running speed. The optimization process involves defining a fitness function that minimizes the reconstruction error, quantified as the Root Mean Square Error (RMSE) between the original noisy time series and the sum of the reconstructed modes obtained from VMD. This approach ensures that the selected parameters effectively balance decomposition accuracy and noise reduction. The parameter K was optimized within the range of 2 to 10, to avoid under-decomposition, which could miss critical signal components, or over-decomposition, which might introduce unnecessary noise components. For the balancing parameter α, the range was set from 500 to 3000, allowing the algorithm to balance between retaining high-frequency components, which could preserve noise, and focusing on low-frequency components, which might oversmooth the signal. Through iterative adjustments within these ranges, TSO identifies the optimal parameters that achieve the lowest RMSE, enhancing the denoising performance of VMD. As shown in Fig 4, the optimized VMD algorithm successfully decomposes the running speed sequence into three modal components, where high-frequency noise is captured in components 2 and 3, and component 1, which has the highest similarity to the original speed sequence, represents the denoised running speed curve..

### BILSTM-AEAM algorithm for time series information extraction

**Fig 5:**
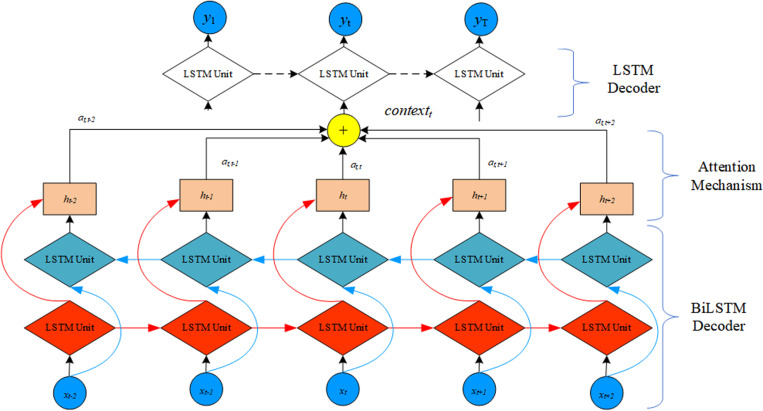
Diagram of BILSTM-AEAM algorithm structure.

Based on the classical LSTM-AE model, this paper designs a sliding window attention mechanism that integrates BILSTM and proposes the BILSTM-AEAM algorithm for reconstructing feature vector curves. The model structure, as shown in Fig 5, consists of three parts: the BILSTM encoder, the attention mechanism, and the LSTM decoder.

#### (1) BILSTM encoder.

In this paper, the BILSTM unit is used to implement the encoder of the LSTM-AE model, which learns the temporal patterns of the feature vector sequence both forward and backward, thereby enhancing the encoding capability of the model. The BILSTM encoder encodes the input data *x* =  [*x*_*1*_*, x*_*2*_, …, *x*_*T*_] (where T is the length of the input data sequence x) and produces the output of the encoder’s hidden layer ht. The calculation formula for *h*_*t*_ is as follows:


$${\vec{h}}_{t}=f\left(x_{t},{\vec{h}}_{t-1}\right)$$ (14)

$${\overset{\leftarrow }{h}}_{t}=f\left(x_{t},{\overset{\leftarrow }{h}}_{t-1}\right)$$ (15)

$$h_{t}={\left[{\vec{h}}_{t}^{\text{T}};{\overset{\leftarrow }{h}}_{t}^{\text{T}}\right]}^{\text{T}}$$ (16)

In the formula, ${\vec{h}}_{t}$ and ${\overset{\leftarrow }{h}}_{t}$ represent the outputs of the forward and backward LSTM hidden layers, respectively. The function f is a nonlinear function in the LSTM unit of the encoder that maps the input to the output of the hidden layer.

#### (2) Attention mechanism.

To improve the quality of the input data for the decoder, a global attention mechanism is introduced, which uses the hidden layer output information of the encoder at all time steps to construct the input for the decoder, allowing for the exploration of temporal relationships on a global scale. However, this approach has issues such as an excessive number of training parameters and redundant information in the hidden layer outputs. Extracting key hidden layer outputs from the LSTM unit of the encoder using L2 regularization to construct the encoder input increases the time complexity, leading to a sharp rise in training time.

The autoencoder relies most on the hidden layer output information of the encoder before and after time t when reconstructing the input at time t (*x*_*t*_). To address the issues with the above attention mechanism, this paper designs a sliding window attention mechanism that integrates BILSTM. It uses a sliding window to encircle the hidden layer output information of the BILSTM encoder at N time steps before and after time t, combined with the hidden layer output information of the LSTM decoder at the previous time step (st-1) to construct the input for the decoder at time t (context). The calculation formula is as follows:


$$e_{tj}=V_{a}\tanh\left(W_{a}s_{t-1}+U_{a}h_{j}\right)$$ (17)

$$a_{tj}=\frac{\exp\left(e_{tj}\right)}{\sum_{j=t-2}^{t+2}\exp\left(e_{tj}\right)}$$ (18)

$${\text{context}}_{t}=\sum_{j=t-N/2}^{t+N2}a_{tj}h_{j}$$ (19)

In the formula, *Va*, *Wa*, and *Ua* are weight matrices, *e*_*tj*_ is the initial attention coefficient required to construct context_*t*_, and *a*_*tj*_ is the normalized attention coefficient. Within *N* time steps, the weighted summation of *a*_*tj*_ and *h*_*j*_ yields the input for the decoder, *conext*_*t*_. The value of *a*_*tj*_ ranges from (0, 1) and satisfies the following condition:


$$\sum_{j=t-N/2}^{t+N/2}a_{tj}=1$$ (20)

Regarding the selection of the sliding window N, this paper uses a dataset of normal sample sequences of current, voltage, and running speed to set different sliding window sizes N and train the BILSTM-AEAM algorithm. Experimental results show that when N is 5, the reconstruction error of each feature sequence is minimized, resulting in the best training effect. Therefore, this paper sets the sliding window size of the BILSTM-AEAM algorithm to 5.

This paper proposes a sliding window attention mechanism that integrates BILSTM. On the one hand, it uses a sliding window centered at time t to encircle the encoder’s hidden layer outputs for N time steps, eliminating hidden layer information that is irrelevant to the reconstruction of x_*t*_. On the other hand, the bidirectional mining function of BILSTM ensures that the hidden layer output h_*t*_ contains bidirectional temporal information of the input data, allowing the sliding window of size N to contain information equivalent to 2 ×  N. Compared to the two attention mechanisms mentioned above, the advantage of the attention mechanism proposed in this paper is that it fully encompasses the key information needed for the reconstruction of x_*t*_ with a minimal increase in training parameters, significantly improving the quality of the decoder’s input data while maintaining a fast model training speed.

#### (3) LSTM decoder.

The decoder part uses an LSTM unit to decode context_*t*_, obtaining the reconstructed data y_*t*_ for x_*t*_. The formula is as follows:


$$s_{t}=g\left(s_{t-1},{\text{contex}}_{t}\right)$$ (21)

$$y_{t}=W_{l}s_{t}+u_{l}$$ (22)

In the formula, g is the nonlinear function in the LSTM unit of the decoder that maps the input to the output of the hidden layer. *W*_*l*_ and *u*_*l*_ are the parameter matrix and constant coefficient of the fully connected layer, respectively.

In this study, RMSE (Root Mean Square Error) is a key metric used to evaluate the reconstruction error between the original and reconstructed data by the LSTM-AE model. It measures how accurately the model captures essential patterns in elevator operation data. A lower RMSE indicates better feature extraction, which enhances the model’s ability to differentiate between normal and faulty conditions. By minimizing RMSE, the model’s predictive accuracy and robustness in classifying elevator fault precursors are improved. This directly impacts the performance of the SoftMax classifier, which uses these reconstruction errors to predict faults, thereby supporting effective predictive maintenance. The reconstruction error RMSE is calculated using the original data $x=\left[x_{1},x_{2},\cdots ,x_{T}\right]$ and the reconstructed data $y=\left[y_{1},y_{2},\cdots ,y_{T}\right]$, with the formula as follows[24–26]:


$$RMSE=\sqrt{\frac{1}{T}\sum_{i=1}^{T}{\left(x_{i}-y_{i}\right)}^{2}}$$ (23)

The prediction of precursors is achieved by integrating the reconstruction errors of various elevator features through a SoftMax classifier. Taking the precursor of overspeed current fault as an example, it is input into the elevator operation and maintenance knowledge base system. The cause of the fault is determined as the main circuit output grounding or short circuit, and the encoder signal is incorrect through phenomenon and rule matching. The treatment measures include checking whether the motor is short-circuited or grounded and whether the encoder-related wiring is correct and reliable, ultimately achieving predictive maintenance of the elevator.

#### (4) Cross-validation.

To ensure the robustness and reliability of the proposed method, rigorous statistical validation techniques were employed throughout the study. Cross-validation was used as a primary method to evaluate the generalizability of the model. Specifically, a k-fold cross-validation approach was implemented, where the data set was divided into *k* subsets. The model was trained on *k* − 1 subsets and validated on the remaining subset. This process was repeated *k* times, with each subset serving as the validation set once, ensuring that every data point contributed to both training and validation. The cross-validation error $E_{cv}$ is calculated as follows:


$$E_{cv}=\frac{1}{k}\sum_{i=1}^{k}E_{i}$$ (24)

where $E_{i}$ is the error on the *i*^*-th*^ validation set. This formula allows for an averaged estimation of the model’s performance across all folds, providing a more reliable measure of its generalizability.(In the study, k is set to 5.)

#### (5) Shapley additive explanations.

In this section, we use Shapley Additive Explanations (SHAP) values to assess the importance of features such as current, voltage, and running speed in predicting elevator fault precursors. SHAP values provide a unified measure of feature importance by assigning an individual contribution value to each feature for each prediction. This method is based on cooperative game theory and offers a fair allocation of the “payout” (prediction) among features (players).

The SHAP value for a feature is computed by considering all possible combinations of features and evaluating the marginal contribution of that feature across these combinations. Mathematically, the SHAP value ϕ i for feature i is defined as:


$$\phi_{i}=\sum_{S\subseteq F\setminus \{i\}}\frac{|S|!(|F|-|S|-1)!}{|F|!}[f(S\cup \{i\})-f(S)]$$ (25)

Where *S* is a subset of all features *F* excluding feature *i*. |S| is the number of features in subset *S*. *f*(*S*) is the prediction value based on the subset of features *S*. *f*(*S* ∪ {*i*}) is the prediction value when feature i is added to subset S.

**Fig 6:**
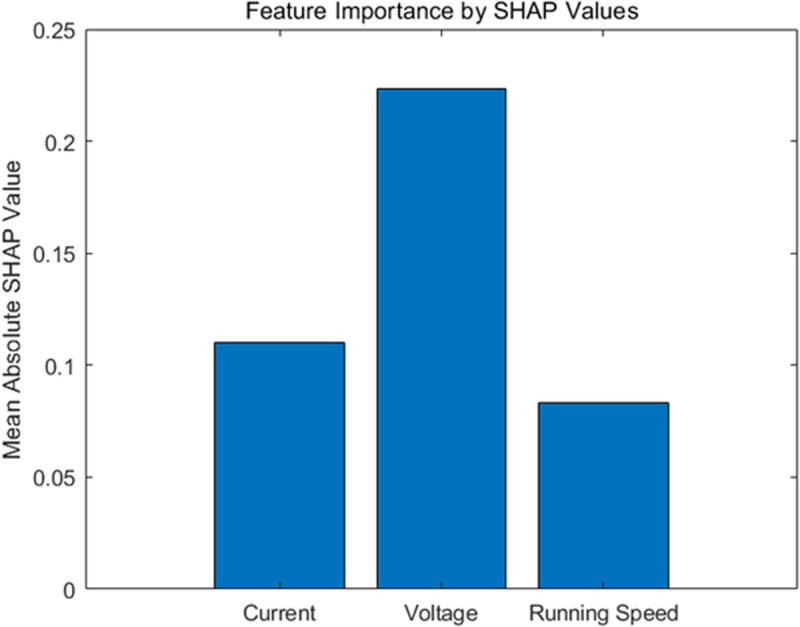
Feature importance ranking.

Applying SHAP values allows us to rank the features based on their average contribution to the model’s predictions. The analysis showed that among the selected features, running speed had the highest average SHAP value, indicating its dominant role in the prediction model. Voltage and current also contributed significantly, but to a lesser extent compared to running speed, as show in Fig 6.

## Results and analysis

### Experimental environment and sample dataset

The experiment in this paper uses Python 3.6 to build the network model. By parsing the elevator operation log files from an Internet of Things (IoT) platform of a company in Nantong City, Jiangsu Province, China, data on elevator current, voltage, frequency, running speed, and acceleration were obtained. The data collection period spanned from June 1, 2023, to September 30, 2023. The dataset includes operational data from 10 elevators, with each parameter providing approximately 1000 data points per day. The ACDR method was used to preliminarily select the feature vector set of current, voltage, and running speed for elevator fault precursor prediction.

**Fig 7:**
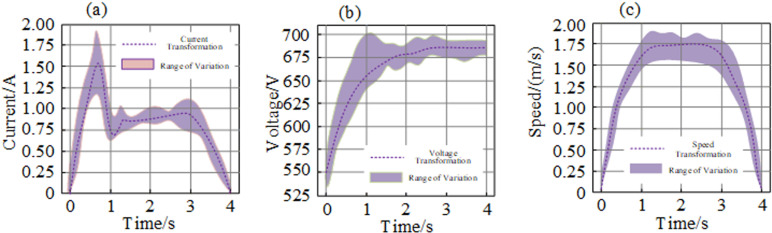
Variation range diagram of elevator current, voltage and speed under normal operation. (a). Current; (b) voltage; (c) speed.

The relationship between elevator current, voltage, and running speed parameters and fault information during the ascending and descending processes was analyzed, taking the ascending process from the 2nd to the 13th floor and the descending process from the 13th to the 2nd floor as examples. As shown in Fig 7, under normal conditions, the time series data curves of voltage during the ascending process, and current and running speed during the descending process, remain stable within a small range of changes. The time series data of voltage during the ascending process, and current and running speed during the descending process, were used as samples. The time series data of each parameter within the normal range of changes were defined as normal samples.

Regarding abnormal samples, an instance is classified as abnormal if at least one parameter’s running curve—whether voltage during the ascending process, current during the descending process, or running speed—deviates beyond the established normal range prior to the occurrence of four specific fault types, including overspeed current and overspeed voltage. Consequently, the time series data of each parameter that lies outside the normal range until a fault occurs is labeled as an abnormal sample for that particular fault.

Analysis of the elevator operation data reveals that abnormal samples related to overspeed, constant speed, and acceleration current faults are relatively more frequent. This is largely due to common issues such as excessive elevator load or short circuits caused by grounding at the motor wiring end. Conversely, faults like abnormal braking unit behavior and excessively high input voltage are less frequent, leading to fewer abnormal samples for overspeed voltage faults. To address sample imbalance, the existing samples were augmented based on the underlying fault mechanisms, thereby constructing a more balanced experimental dataset, as detailed in Table 1.

**Table 1:** Experimental dataset.

Sample Type	Fault Category	Preexpansion Sample Size	Post-expansion Sample Size
Normal Samples	0	2,100	2,100
Overspeed Current Abnormal Samples	1	150	296
Constant Speed Current Abnormal Samples	2	170	260
Acceleration Current Abnormal Samples	3	140	300
Overspeed Voltage Abnormal Samples	4	40	245

### Experimental results analysis

#### (1) Comparison of model training speed and generalization ability before and after improvement.

**Fig 8:**
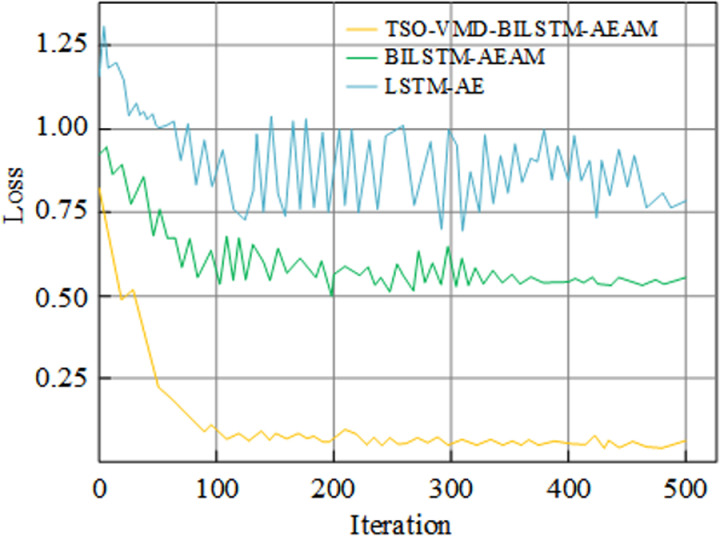
Comparison of training results before and after improvement of LSTM-AE algorithm.

The training and test sets are obtained from the experimental dataset in Table 1 in a 4:1 ratio. To verify the training effect of the improved algorithm, three algorithms before and after improvement are selected for comparative analysis, as shown in Fig 8. The increased complexity of the improved LSTM-AE algorithm, specifically the TSO-VMD-BILSTM-AEAM, involves additional components such as Bidirectional LSTM and an attention mechanism. While these enhancements result in a longer average iteration time of 21 seconds compared to the 9 seconds of the basic LSTM-AE model, the overall training efficiency is significantly improved due to the reduction in the number of training iterations. Specifically, the improved algorithm stabilizes the training loss after just 130 rounds compared to the 440 rounds required by the original LSTM-AE model. This reduction in iterations outweighs the increased time per iteration, resulting in a net reduction in total training time from 66 minutes to 45 minutes, which corresponds to a 32% improvement in training speed. This demonstrates that the increased model complexity, although slightly raising iteration time, ultimately does not negatively impact the overall training efficiency due to the more rapid convergence of the improved model.

**Fig 9:**
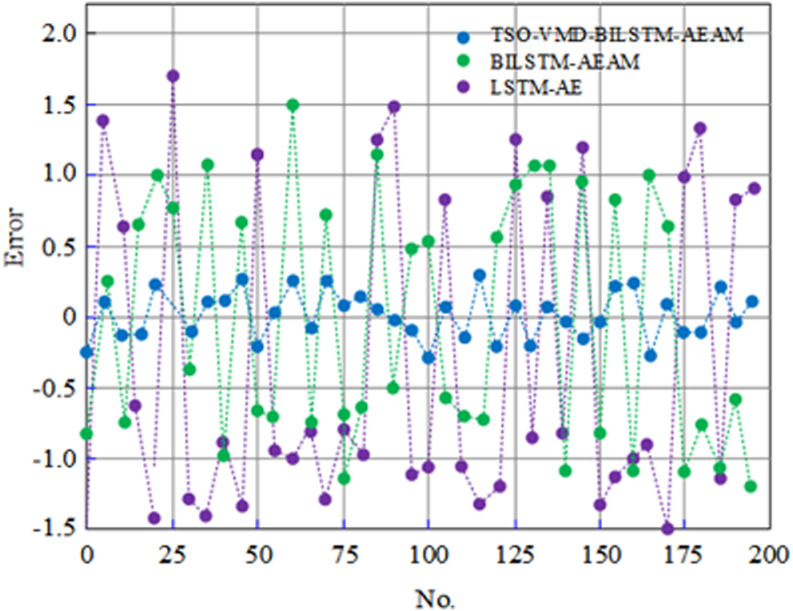
Comparison of test errors before and after improvement of LSTM-AE algorithm.

The reconstruction error represents the degree of deviation of elevator operation parameters from normal operation data, and the generalization ability of the algorithm can be assessed by the test error. To compare the generalization ability of the models, this paper analyzes the test errors of the LSTM-AE algorithm before and after improvement, as shown in Fig 9.

Fig 9 demonstrates that the absolute test errors for most samples of the LSTM-AE algorithm exceed 0.9, whereas the BILSTM-AEAM algorithm shows test errors ranging primarily between 0.4 and 1.1. In contrast, the improved LSTM-AE algorithm, which integrates the VMD method, BILSTM units, and an attention mechanism, consistently maintains absolute test errors below 0.3, indicating superior generalization ability.

#### (2) Comparative analysis of accuracy of different models.

To evaluate the efficacy of the improved LSTM-AE algorithm for fault precursor prediction, experiments were conducted using various models, including GRU, LSTM, LSTM-AE, EMD-LSTM-AE, and the proposed improved LSTM-AE algorithm. The performance of these models was assessed using TPR (True Positive Rate), FPR (False Positive Rate), and AUC (Area Under Curve) as key evaluation metrics for classification accuracy. During the initial development of the elevator operation and maintenance knowledge base, the scarcity of abnormal data resulted in an imbalanced ratio of normal to abnormal samples in the test set. The AUC index offers an advantage in this context as it combines the TPR for normal samples and the FPR for abnormal samples, thus providing a balanced evaluation of the model’s predictive performance, even under conditions of sample imbalance. To further strengthen the robustness of the experimental results, the study employed ten test sets, each with an equal total number of samples but differing in the proportion of normal to abnormal samples, as detailed in Table 2.

**Table 2.** Ten test sets of data.

Sample	NO. 1	NO. 2	NO. 3	NO. 4	NO. 5	NO. 6	NO. 7	NO. 8	NO. 9	NO. 10
Number of Normal Samples	20	40	60	80	100	120	140	160	180	190
Number of Abnormal Samples	180	160	140	120	100	80	60	40	20	10

**Fig 10:**
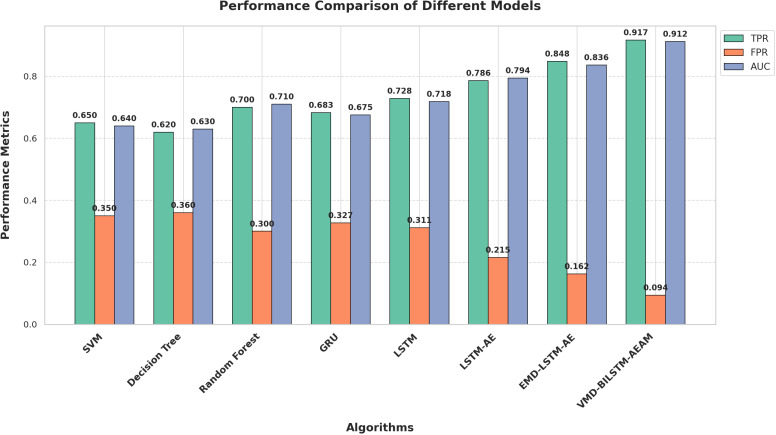
Comparison of TPR, FPR, and AUC for Different Methods (Five-Fold Cross-Validation).

Use the above 10 sets of data as the test set for the aforementioned six algorithms, and calculate the mean values of TPR, FPR, and AUC indices for each algorithm. The results are shown in Fig 10.

From Fig 10, it can be seen that traditional models like SVM and Decision Tree have lower True Positive Rates (TPR) and higher False Positive Rates (FPR) compared to more advanced models. The Random Forest model shows some improvement but still falls short of the performance of deep learning models. The LSTM-AE algorithm, compared to GRU and LSTM networks, achieves a higher TPR and a lower FPR, making it more effective for elevator fault precursor prediction tasks. The EMD-LSTM-AE algorithm further enhances performance with significant improvements in both TPR and AUC due to its denoising capabilities. The proposed VMD-BILSTM-AEAM algorithm outperforms the traditional LSTM-AE algorithm, showing a 13.1% increase in TPR and a 12.1% decrease in FPR. Furthermore, the AUC value of 0.912 is higher than all other models in the comparison, demonstrating the superiority of this method in elevator precursor prediction. The VMD-BILSTM-AEAM algorithm not only improves the accuracy of fault detection but also significantly reduces false alarms, which is crucial for the reliable operation and maintenance of elevators. This robust ability to handle and analyze time-series data, even in noisy environments, highlights its potential for broader applications in other domains requiring accurate and timely fault detection.

To validate the robustness of the VMD-BILSTM-AEAM model, statistical analysis was conducted, including confidence interval calculations for key performance metrics: TPR (True Positive Rate), FPR (False Positive Rate), and AUC (Area Under Curve). The variability and reliability of the results were assessed through 20 similar experiments, as shown in Table 3. The mean TPR was 0.919 with a 95% confidence interval of 0.915 to 0.924, indicating stable performance in correctly identifying positive cases. The mean FPR was 0.090 with a 95% confidence interval of 0.087 to 0.092, reflecting consistent performance in minimizing false positives. The AUC had a mean value of 0.919 with a confidence interval of 0.915 to 0.923, demonstrating high accuracy and stability in classifying elevator fault precursors. These narrow confidence intervals suggest that the model’s performance metrics are highly reliable and consistent, showing little sensitivity to data variations, thus validating the effectiveness and robustness of the proposed method. This analysis confirms the consistent performance of the model across multiple experiments, further supporting the statistical credibility of the results.

**Table 3.** Confidence interval summary.

Metric	Mean	CI Lower Bound	CI Upper Bound
TPR	0.919	0.915	0.924
FPR	0.09	0.087	0.092
AUC	0.919	0.915	0.923

## Conclusions

This paper proposes an enhanced LSTM-AE algorithm for predicting elevator fault precursors, integrating the TSO-optimized Variational Mode Decomposition (VMD) and a sliding window attention mechanism that incorporates Bidirectional Long Short-Term Memory (BILSTM). The improvements aim to enhance the accuracy and efficiency of elevator fault predictions. Based on the experimental analysis, the following updated conclusions are drawn:

(1) Noise Resistance and Data Quality Improvement: The TSO-optimized VMD algorithm significantly enhances the noise resistance of the improved LSTM-AE algorithm. This results in better data quality and provides more accurate input data for fault prediction, ensuring that the model can effectively handle the noisy, real-world elevator operation data.
(2) Enhanced Feature Extraction: The sliding window attention mechanism that integrates BILSTM improves the model’s encoding capabilities for time series data. By focusing on the most relevant information to construct attention weights, this mechanism enhances the quality of the decoder’s input data, which in turn boosts the overall predictive performance of the model.
(3) Increased Prediction Accuracy and Efficiency: The improved LSTM-AE algorithm demonstrates significantly enhanced training speed and generalization ability, with a 13.1% increase in the True Positive Rate (TPR) and a 12.1% reduction in the False Positive Rate (FPR). This improvement effectively predicts elevator fault precursors in advance, providing a robust tool for predictive maintenance and demonstrating a new approach in elevator fault management.
(4) Application in Predictive Maintenance: The predictive method proposed in this paper utilizes the prediction results as fault precursors within the elevator operation and maintenance system. By matching the predicted results with known fault phenomena and rules, the model determines the likely cause of faults and recommends appropriate treatment plans, enabling proactive and predictive maintenance based on early detection of operational parameter deviations.
(5) Generalization Across Different Domains: The VMD-BILSTM-AEAM algorithm demonstrates strong generalization capabilities beyond elevator fault prediction. Since the model is trained using time-series data with noise-filtering and feature-selection techniques, it can be effectively applied to other predictive maintenance tasks involving sequential sensor data. For instance, in industrial equipment monitoring, the proposed method can identify early warning signs of motor failures, hydraulic system malfunctions, or anomalies in automated production lines. Similarly, in smart building management, this model can be adapted to predict HVAC (Heating, Ventilation, and Air Conditioning) system failures or energy consumption patterns. Additionally, in intelligent transportation systems, the model can be leveraged to detect abnormal vehicle behavior or predict railway component failures.

The proposed VMD-BILSTM-AEAM algorithm not only achieves higher predictive accuracy but also demonstrates strong adaptability to real-world applications where real-time and high-precision fault detection is essential. The trade-off between increased computational complexity and improved accuracy is justified by the model’s superior performance in identifying early fault precursors, reducing false alarms, and enabling more reliable predictive maintenance. This balance ensures that the model remains practical for deployment in critical scenarios such as elevator monitoring, where safety and operational efficiency are paramount.

Although the proposed VMD-BILSTM-AEAM algorithm introduces additional computational complexity due to the integration of Bidirectional LSTM units and an attention mechanism, its advantages in real-world applications outweigh the cost of increased processing time. In scenarios where predictive maintenance is crucial—such as elevator fault detection in high-rise buildings, industrial automation, and transportation systems—minimizing false alarms and accurately identifying early fault precursors are of paramount importance.

The increased complexity of the model enhances feature extraction from noisy sensor data, leading to more reliable predictions. For instance, real-time monitoring in high-traffic commercial buildings requires highly accurate fault detection systems to prevent unexpected breakdowns. A model with lower predictive accuracy but faster execution time may generate excessive false positives or negatives, resulting in unnecessary maintenance costs or, conversely, unanticipated failures.

Although the average iteration time of the improved model has increased from 9 seconds to 21 seconds, the overall training time has been reduced by 32% due to faster convergence (130 iterations vs. 440 iterations for the standard LSTM-AE model). More importantly, once the model is trained, real-time prediction of elevator faults is highly efficient, as the inference process remains computationally lightweight. Thus, this trade-off between complexity and accuracy significantly enhances the model’s practical usability in real-world applications where precision is critical.

In summary, this study introduces an advanced fault precursor prediction model that integrates VMD for noise reduction, BILSTM for sequence learning, an attention mechanism for feature prioritization, and SHAP for interpretability. The core advantage of this model lies in its ability to process complex, real-world elevator operation data and extract meaningful insights while maintaining transparency in decision-making.

By providing a more intuitive breakdown of our methodology, we aim to bridge the gap between theoretical advancements and real-world implementation. The proposed model is not only highly accurate in predicting faults but also readily interpretable, allowing engineers and maintenance personnel to confidently apply it in practical scenarios. Future work will focus on further enhancing model explainability and adaptability across multiple industrial domains.

## Supporting information

S1 Data Data and code. (ZIP)
