# Supplementary material for: Elevator fault precursor prediction based on improved LSTM-AE algorithm and TSO-VMD denoising technique
Source: PLoS One. 2025 Apr 24;20(4):e0320566. doi: 10.1371/journal.pone.0320566 (PMC12021203; doi:10.1371/journal.pone.0320566)
Supplement: S1 Data — (ZIP) [file pone.0320566.s001.zip › Data and code/Code for Elevator Fault Prediction.docx]

数据预处理与特征选择
import numpy as np

import pandas as pd

from sklearn.preprocessing import StandardScaler

# 加载电梯运行数据

data = pd.read_csv('elevator_data.csv') # 将文件路径替换为实际数据文件路径

# 特征选择：基于ACDR算法选择相关特征

def acdr_feature_selection(data):

"""

ACDR算法进行特征选择，筛选出电流、电压和运行速度等关键特征。

"""

# 计算特征相关性和区分度，选择重要特征

features = ['current', 'voltage', 'speed'] # 假设这些特征是通过ACDR选择出来的

selected_features = data[features] # 根据ACDR结果选择特征

return selected_features

# 应用特征选择

selected_data = acdr_feature_selection(data)

# 数据标准化

scaler = StandardScaler()

scaled_data = scaler.fit_transform(selected_data)

TSO优化的VMD算法进行降噪处理：

from vmdpy import VMD # 确保安装了vmdpy库，或者使用自定义VMD实现

import random

# TSO-VMD：使用TSO优化VMD的参数

def tso_vmd(signal, alpha=2000, tau=0, K=3, DC=0, init=1, tol=1e-7):

"""

使用金枪鱼群优化算法（TSO）优化VMD参数，并进行降噪。

"""

# TSO优化过程（此处为简化示例，实际应包括TSO算法实现）

# 例如，优化参数alpha、tau和K等

def optimize_vmd_params(signal):

# 初始化VMD参数

best_alpha = alpha

best_tau = tau

best_K = K

# 进行若干次迭代以优化参数（TSO实现细节略）

for _ in range(50): # 假设进行50次迭代

# 随机调整参数并评估效果

alpha_try = random.uniform(1500, 2500)

K_try = random.randint(2, 5)

# 假设一个简单的评价指标函数evaluate_vmd效果

_, _, omega = VMD(signal, alpha=alpha_try, tau=best_tau, K=K_try, DC=DC, init=init, tol=tol)

# 假设通过评估函数得到更优参数

if np.mean(omega) > np.mean(best_K): # 这里用均值作为评价（示例）

best_alpha = alpha_try

best_K = K_try

return best_alpha, best_tau, best_K

# 优化VMD参数

optimized_alpha, optimized_tau, optimized_K = optimize_vmd_params(signal)

# 应用优化后的VMD进行信号分解和降噪

u, u_hat, omega = VMD(signal, alpha=optimized_alpha, tau=optimized_tau, K=optimized_K, DC=DC, init=init, tol=tol)

return u

# 对运行速度数据应用TSO-VMD进行降噪处理

denoised_speed = tso_vmd(scaled_data[:, 2]) # 假设速度数据在第三列

改进的LSTM-AE模型构建和训练

import torch

import torch.nn as nn

import torch.optim as optim

from torch.utils.data import DataLoader, TensorDataset

# 定义改进的LSTM-AE模型，包含BiLSTM编码器和注意力机制

class BiLSTM_AE(nn.Module):

def __init__(self, input_dim, hidden_dim, output_dim, n_layers):

super(BiLSTM_AE, self).__init__()

# BiLSTM编码器部分

self.encoder = nn.LSTM(input_dim, hidden_dim, n_layers, batch_first=True, bidirectional=True)

# 注意力机制层，用于构建解码器输入

self.attention = nn.Linear(hidden_dim * 2, 1)

# LSTM解码器部分

self.decoder = nn.LSTM(hidden_dim * 2, output_dim, n_layers, batch_first=True)

def forward(self, x):

# 编码过程

encoded, (hn, cn) = self.encoder(x)

# 注意力机制计算

attention_weights = torch.softmax(self.attention(encoded), dim=1)

context = attention_weights * encoded

context = context.sum(dim=1) # 加权求和

# 解码过程

decoded, _ = self.decoder(context.unsqueeze(1))

return decoded

# 设置模型参数

input_dim = scaled_data.shape[1] # 输入维度为特征数

hidden_dim = 64 # 隐藏层维度

output_dim = input_dim # 输出维度等于输入维度

n_layers = 2 # LSTM层数

batch_size = 32 # 批处理大小

learning_rate = 0.001 # 学习率

n_epochs = 100 # 训练轮数

# 将数据转换为PyTorch张量

X = torch.tensor(np.array(denoised_speed).reshape(-1, 1, len(denoised_speed)), dtype=torch.float32) # 假设是一维时序数据

dataset = TensorDataset(X, X) # 自编码器的目标是重构输入

dataloader = DataLoader(dataset, batch_size=batch_size, shuffle=True)

# 实例化模型、损失函数和优化器

model = BiLSTM_AE(input_dim, hidden_dim, output_dim, n_layers)

criterion = nn.MSELoss() # 均方误差损失

optimizer = optim.Adam(model.parameters(), lr=learning_rate)

# 模型训练过程

for epoch in range(n_epochs):

model.train()

for batch_X, _ in dataloader:

optimizer.zero_grad() # 清零梯度

output = model(batch_X) # 模型前向传播

loss = criterion(output, batch_X) # 计算损失

loss.backward() # 反向传播

optimizer.step() # 更新参数

# 输出训练过程中的损失

print(f'Epoch {epoch + 1}/{n_epochs}, Loss: {loss.item()}')

模型预测与评估
# 模型评估函数

def evaluate_model(model, test_data):

"""

评估模型的性能，计算重构误差。

"""

model.eval() # 设置模型为评估模式

with torch.no_grad():

predictions = model(test_data)

# 计算评估指标，如RMSE

# 这里只是一个示例，实际可根据需要计算TPR、FPR等指标

rmse = torch.sqrt(criterion(predictions, test_data))

print(f'Evaluation RMSE: {rmse.item()}')

return predictions

# 示例评估

test_data = torch.tensor(np.array(denoised_speed[-100:]).reshape(-1, 1, len(denoised_speed[-100:])), dtype=torch.float32) # 使用最后100个样本进行测试

predictions = evaluate_model(model, test_data)
